# Hypoxia induces epithelial-mesenchymal transition via activation of SNAI1 by hypoxia-inducible factor -1α in hepatocellular carcinoma

**DOI:** 10.1186/1471-2407-13-108

**Published:** 2013-03-09

**Authors:** Lin Zhang, Gang Huang, Xiaowu Li, Yujun Zhang, Yan Jiang, Junjie Shen, Jia Liu, Qingliang Wang, Jin Zhu, Xiaobin Feng, Jiahong Dong, Cheng Qian

**Affiliations:** 1 Institute of Hepatobiliary Surgery, Southwest Hospital, Third Military Medical University, Chongqing, China; 2 Institute of Pathology and Southwest Cancer Center, Southwest Hospital, Third Military Medical University, Chongqing, China; 3 Department of Radiology, Southwest Hospital, Third Military Medical University, Chongqing, China; 4 Institute of Hepatobiliary Surgery, General Hospital of PLA, Beijing, 100853, PR China; 5 Department of Medical Genetics, College of Basic Medicine, Third Military Medical University, Chongqing, China

**Keywords:** Epithelial-mesenchymal transition, Hypoxia, Hypoxia-inducible factor-1α, SNAI1, Hepatocellular carcinoma

## Abstract

**Background:**

High invasion and metastasis are the primary factors causing poor prognosis of patients with hepatocellular carcinoma (HCC). However, the molecular mechanisms underlying these biological behaviors have not been completely elucidated. In this study, we investigate the molecular mechanism by which hypoxia promotes HCC invasion and metastasis through inducing epithelial-mesenchymal transition (EMT).

**Methods:**

The expression of EMT markers was analyzed by immunohistochemistry. Effect of hypoxia on induction of EMT and ability of cell migration and invasion were performed. Luciferase reporter system was used for evaluation of Snail regulation by hypoxia-inducible factor -1α (HIF-1α).

**Results:**

We found that overexpression of HIF-1α was observed in HCC liver tissues and was related to poor prognosis of HCC patients. HIF-1α expression profile was correlated with the expression levels of SNAI1, E-cadherin, N-cadherin and Vimentin. Hypoxia was able to induce EMT and enhance ability of invasion and migration in HCC cells. The same phenomena were also observed in CoCl2-treated cells. The shRNA-mediated HIF-1α suppression abrogated CoCl2-induced EMT and reduced ability of migration and invasion in HCC cells. Luciferase assay showed that HIF-1α transcriptional regulated the expression of SNAI1 based on two hypoxia response elements (HREs) in SNAI1 promoter.

**Conclusions:**

We demonstrated that hypoxia-stabilized HIF1α promoted EMT through increasing SNAI1 transcription in HCC cells. This data provided a potential therapeutic target for HCC treatment.

## Background

Metastasis is the main cause of deaths for patients with many solid cancers. Approximately 90% of deaths caused by cancers result from the metastatic spread of primary tumors [1]. Therefore, it is critical to understand the mechanisms of metastasis and to identify new targets for therapy. Recently, two mechanisms of metastasis have received significant attention: (1) epithelial mesenchymal transition (EMT) and mesenchymal epithelial transition (MET) [2-8] and (2) interactions between tumor cells and microenvironment [9-15]. EMT is believed to be a major mechanism by which cancer cells become migratory and invasive. A variety of cancer cells display features of EMT. In addition, multiple steps of metastasis are influenced by the tumor microenvironment which may determine the course and severity of metastasis [16-23] .

Hypoxia is a critical microenvironment in tumor pathogenesis. It occurs in series of distinct steps that include tumor cell invasion, intravasation, extravasation and proliferation. There is a close relationship between hypoxia and tumor metastasis and poor prognosis. Several mechanisms have been proposed to explain how hypoxia might lead to a poor prognosis in the clinical settings, and none of which are mutually exclusive [4,24-27].

This hypoxic response is mainly regulated by the hypoxia-inducible factor 1 (HIF-1), a basic HLH transcription factor composed of two subunits, HIF-1α and HIF-1β. The HIF-1α subunit is regulated by oxygen tension, whereas HIF-1β is constitutively expressed [28-32]. Over-expression of HIF-1α is a common feature of malignant cells and links to poor prognosis in both lymph-node positive [33] and lymph-node negative [34] breast carcinoma. Therefore, the exploration of target genes by HIF-1 may lead to a better understanding of the contribution of hypoxia to tumor progression.

HIF-1α activation correlates with metastasis in many kinds of tumors and promotes metastasis through the regulation of key factors governing tumor cell metastatic potential. E-cadherin is a key molecule related to metastatic potential in the majority of epithelial cancers. It is a cellular adhesion molecule that regulates cell–cell adhesion and stimulates anti-growth signals through interactions with β-catenin in cytoplasm [35]. It has been proposed that HIF-1α mediates repression of E-cadherin expression through the upregulation of E-cadherin-specific repressors Snail and SIP1 [36]. Similarly, hypoxia promotes EMT and metastatic phenotypes in human cancer cells via direct induction of the E-cadherin repressor twist [2].

Hepatocellular carcinoma (HCC) is one of the most common cancers worldwide. Invasion and metastasis in early-stage HCC is an important feature and a crucial unfavorable prognostic factor. Therefore, in this work we investigated how hypoxia could induce EMT and promote metastasis of HCC cells.

## Methods

### Immunohistochemistry

Human liver tissues were obtained from surgical resection specimens of HCC patients in the Institute of Hepatobiliary Surgery, Southwest Hospital, Third Military Medical University. The procedure of human sample collection was approved by the Ethical Committee of Third Military Medical University. A tissue microarray block containing 66 HCC tissues was constructed by using a tissue microarrayer. Immunostaining was performed on tissue microarray slides following the routine protocol. The following antibodies were used: mouse anti-human HIF-1α monoclonal antibody (BD Clontech, USA), mouse anti-human E-cadherin monoclonal antibody, mouse anti-human N-cadherin monoclonal antibody, mouse anti-human Vimentin monoclonal antibody, rabbit anti-human Twist polyclonal antibody and rabbit anti-human SNAI1 polyclonal antibody (Santa Cruz Biotech, USA). Assessment of the staining was based on the percentage of positively stained cells and the staining intensity.

### Cell culture

Human HCC cell lines HepG2 and SMMC-7721 were purchased from Shanghai Cell Collection (Shanghai, China). Human embryonic kidney cell line HEK293 was obtained from Microbix Biosystems (Toronto, ON, Canada). The cells were cultured in Dulbecco’s modified Eagle’s medium supplemented with 10% fetal bovine serum (FBS; GIBCO-BRL) at 37°C under a 5% CO_2_ condition. CoCl_2_ was purchased from Sigma-Aldrich (St. Louis, USA).

### Fluorescent immunostaining

Cells were cultured in 24-well plates at 5 × 10^4^ cells per well. At the indicated time points, media were removed from the cultured cells followed by three washings with PBS. Cells were fixed with 4% polyoxymethylene solution for 20 min and washed with PBS three times. Cells were incubated with primary antibodies and then their corresponding lumophore-conjugated secondary antibodies. DAPI was used for nuclei staining. Finally, cells were observed under a fluorescent microscope or a confocal microscope.

### Adenoviral vector-mediated HIF-1α silencing

The shRNA specifically targeting HIF-1α mRNA was generated by annealing the following primers: Forward: 5'-aGTCGGACAGCCTCACCAAAtttt-3'; Reverse: 5'-aTTTGGTGAGGCTGTCCGACtttt-3', followed by its insertion into pSES-HUS that was digested by *SfiI* to generate HIF-1α siRNA pSES-HUS. After digestion of *PacI*, HIF-1α siRNA pSES-HUS was transfected into *E. Coli BJ5183* with pAdEasy-1 to obtain recombination plasmid pAdeasy-HIF-1α siRNA. After identification, pAdeasy-HIF-1α siRNA was transfected into HEK293 cells for the production of recombinant adenovirus Ad-HIF-1α siRNA (Ad-shHIF-1α). The control adenovirus containing a non-function shRNA (Ad-scrambled) (Forward: 5'-aGACTTCATAAGGCGCATGCtttt-3' Reverse: 5'-aGCATGCGCCTTATGAAGTCtttt-3') is constructed in a similar protocol. The adenoviruses were harvested and purified with the CsCl gradient centrifugation method. The titers of adenoviruses were quantified through TCID_50_ assay on HEK293 cells.

### Quantitative real-time PCR (qRT-PCR)

The SMMC-7721 cells were harvested at the indicated time points. Total RNA was extracted by using Trizol (Invitrogen) according to the manufacturer’s protocol. Reverse transcription was performed according to the protocol of RevertAidTM First Strand cDNA Synthesis Kits (Fermentas). Quantitative PCR was performed by using SYBR premix Ex Taq (TaKaRa) and Applied Biosystems 7300 Real-Time PCR System (Applied Biosystems, USA) supplied with analytical software. The primers used for this study were listed in Additional file 1: Table S6.

### Immunoblotting

Total proteins were separated on 8–12% polyacrylamide gels and transferred onto 0.45 μm nitrocellulose in a buffer containing 25 mmol/L Tris–HCl (pH 8.3), 192 mmol/L glycine, 20% methanol and blocked with 5% fat-free dry milk in PBS for 2 h. The membranes were incubated with primary antibodies, as described in Immunohistochemistry. β-actin was used as internal control.

### Cell migration and invasion assays

The invasion assays were performed using Millicell inserts (Millipore, Billerica, MA, USA) coated with Matrigel (BD Biosciences, Sparks, MD, USA). 2.5 × 10^4^ cells were seeded per upper chambers in serum-free DMEM whereas the lower chambers were loaded with DMEM containing 5% FBS. After 24 hrs, the non-migrating cells on the upper chambers were removed by a cotton swab, and cells invaded through the matrigel layer to the underside of the membrane were stained by crystal violet. The cell numbers were counted. Cell migration assays were performed similarly, but without Matrigel.

### Cell cycle analysis

For identifying cells at different stages of cell cycle, vector infected cells were prepared as a single cell suspension of 1–2 × 10^6^ cells/mL in PBS. After the cells were fixed with pre-cold 70% ethanol for 2 hrs, the cells were washed two times with PBS and were stained with Propidium Iodide (PI) at the final concentration of 50 μg/mL with RNase at 20 μg/mL in PBS. Treated cells were then evaluated by FACS analysis.

### Colony formation assay

Colony formation assay was performed by using monolayer culture. Cells were plated in a 6-well plate and then cultured under hypoxic condition. Colonies (>50 cells/colony) were counted after staining with crystal violet solution. All the experiments were performed in triplicate wells three times.

### Luciferase reporter vector construction

We used genomic DNA of human normal liver as template to amplify the promoter of SNAI1 gene. The sequences of primer sets were provided upon requested. The PCR products were digested by *KpnI* and *XhoI*, followed by being inserted into pGL3-basic. The resulting plasmids harboring various lengths of SNAI1 promoter were transfected into CoCl_2_-treated SMMC-7721 cells. The activity of luciferase was examined at the indicated time points.

### Statistical analysis

Each experiment was performed at least two times. All values were presented as means ± SD. The statistics was analyzed by unpaired, two-tailed *t*-test. Data were considered to be statistically significant when p < 0.05 (*) and p < 0.01 (**).

## Results

### Expression of hypoxia and EMT related genes in human HCC

In order to know whether hypoxia status is related to EMT in HCC, we firstly investigated expression levels of HIF-1α, HIF-2α, SNAI1, Twist, E-cadherin, N-cadherin and Vimentin in a tissue array containing 66 HCC samples from human patients by immunohistochemistry. HIF-1α and SNAI1 expression was detected in 65% (43/66) and 59% (39/66) of tumor samples, respectively. Coexistence of HIF-1α and SNAI1 was observed in 50% of the cases (33/66) and their expression level was significantly positively correlated (P < 0.01) (Additional file 1: Table S1). In addition, we observed the significant correlations between HIF-2α and SNAI1 and Twist (Additional file 1: Table S1). We also compared the expression profiles between EMT markers and HIF-1α as well as SNAI1. In the HIF-1α positive samples of HCC patients (n = 43), expression of E-cadherin and N-cadherin was found in 10 and 34 samples, respectively. There was a significant negatively correlation in expression level between HIF-1α and E-cadherin (P < 0.01) and positive correlation between HIF-1α and N-cadherin (P < 0.01) (Additional file 1: Table S2). Analysis on SNAI1 expression also showed its correlation with EMT markers in these HCC samples (P < 0.01) (Additional file 1: Table S2). Our data also showed that expression of E-cadherin was significant negative correlated to the expression of N-cadherin and Vimentin (P < 0.01) (Additional file 1: Table S3).

### Overexpression of HIF-1α and SNAI1 in HCC predicts poor prognosis

**Figure 1:**
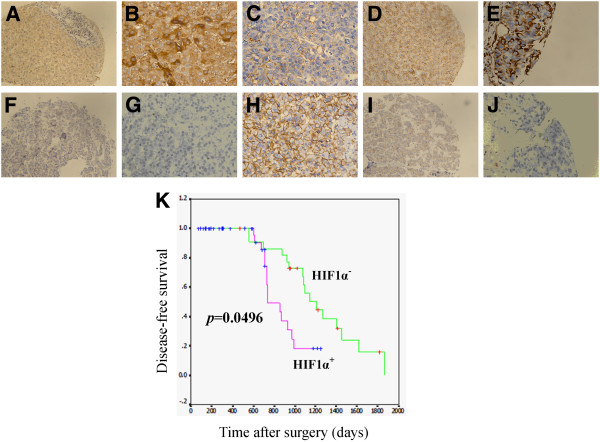
**Overexpression of HIF-1α in HCC is correlated with the level of SNAI1 and EMT markers and predicts poor prognosis.** Immunohistochemistry was performed for determining the expression profile of various proteins, including HIF-1α (**A**, **F**), SNAI1 (**B**, **G**), E-cadherin (**C**, **H**), N-cadherin (**D**, **I**) and Vimentin (**E**, **J**), on HIF-1α^+^ (**A**-**E**) and HIF-1α^-^ (**F**-**J**) HCC samples (**A**, **D**, **F**, **I**: ×200; **B**, **C**, **E**, **G**, **H**, **J**: ×400). (**K**) Disease-free survival after surgery was compared between HIF-1α positive (n = 43) and HIF-1α negative (n = 23) HCC patients.

Overexpression of HIF-1α and SNAI1 in HCC samples were shown to be correlated with pathological classification, TNM staging and tumor volume (P < 0.05). In addition, HIF-1α expression profile was also correlated with severity of cirrhosis (P < 0.05) (Additional file 1: Table S4). Consistent to previous studies, EMT phenotype in HCC samples was found to be significantly correlated with pathological classification, TNM staging, numbers of tumor nodule and tumor size (Additional file 1: Table S5). Prognosis of the HCC patients with HIF-1α expression level was also investigated in our work. Our data showed that disease-free survival was shorter in HIF-1α positive group (n = 43, 824 days) compared with HIF-1α negative group (n = 23, 1144 days, P = 0.0496) (Figure 1). This data suggest that HIF-1α is correlated with SNAI1 expression and EMT phenotype of HCC samples and can predict poor prognosis of HCC patients after surgery.

### Hypoxia induced EMT of HCC cells while reversion occurred under reoxygenation

**Figure 2:**
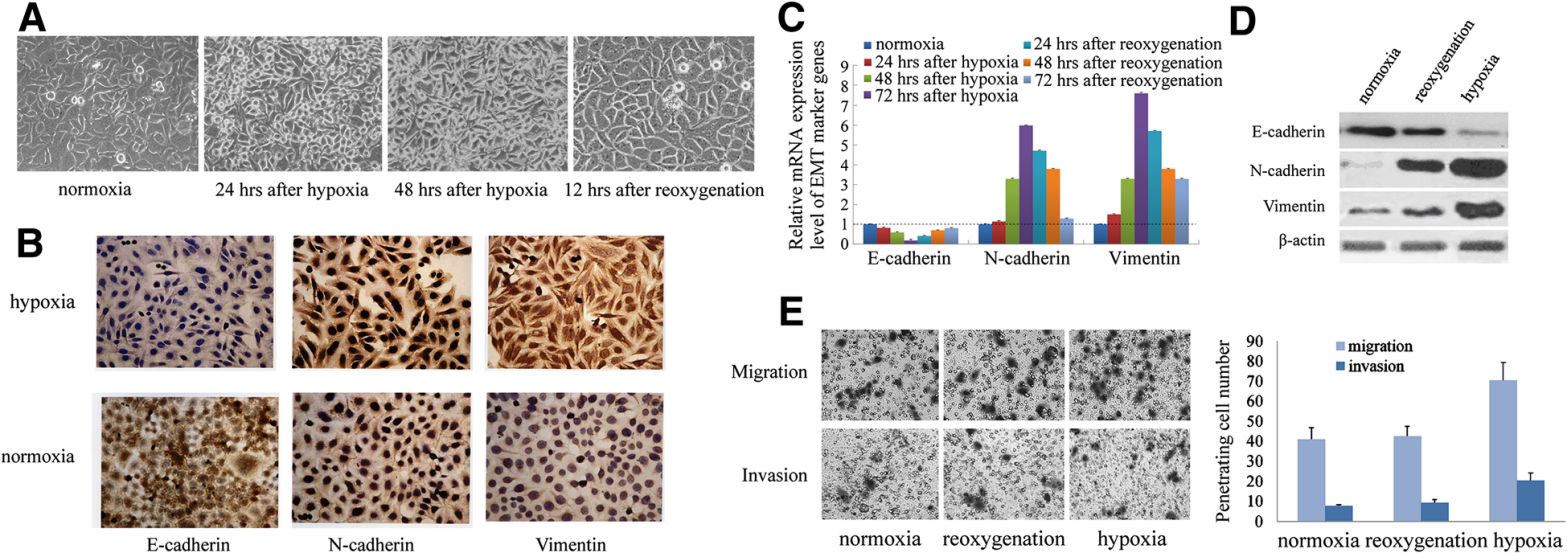
**Hypoxia induced EMT of HCC cells while reversion occurred under reoxygenation.** (**A**) Morphological changes of hypoxia and reoxygenation-treated SMMC-7721 cells were recorded by light microscope (×200). (**B**) Immunohistochemical analysis of E-cadherin, N-cadherin and Vimentin expression was performed on both hypoxically and normoxically cultured SMMC-7721 (×200). (**C**) mRNA levels of E-cadherin, N-cadherin and Vimentin in SMMC-7721 was determined under the conditions of normoxia, hypoxia and reoxygenation by qPCR. GAPDH was used as endogenous reference and mRNA level of SMMC-7721 under normoxia was used as control. Data were shown as mean ± SD of three independent experiments. (**D**) Protein expression of E-cadherin, N-cadherin and Vimentin in SMMC-7721 cells undergoing normoxia, hypoxia and reoxygenation was determined by immunoblotting. β-actin was used as endogenous control. (**E**) The numbers of invasive and migrating SMMC-7721 cells undergoing normoxia, hypoxia and reoxygenation was calculated with crystal violet staining. The average numbers of ten random microscopic fields (×400) was recorded in each experiment. Data was shown as mean ± SD of three independent experiments. Representative images of each group were shown.

The correlation between overexpression of HIF-1α and EMT induction in HCC tissues suggested that hypoxia may regulate the EMT of HCC cells. Therefore, we investigated induction of EMT by hypoxia. We observed morphological changes with characteristic of obtaining mesenchymal phenotype and losing epithelial feature in HCC cell lines under 1% O_2_ condition. When cells were returned to normoxic conditions, its epithelial morphology was regained (Figure 2A and Additional file 2: Figure S1). Immunohistological staining showed that E-cadherin protein was lost in majority of HCC cells, whereas expressions of N-cadherin and Vimentin were extensively detected in these cells under hypoxia (Figure 2B). Immunofluorescent staining also confirmed the enhanced expression of N-cadherin and Vimentin in HCC cells under hypoxia (Additional file 3: Figure S2).

Subsequently, we used qRT-PCR to quantify mRNA levels of EMT markers. Expression of E-cadherin was gradually suppressed when HCC cells were hypoxically cultured. Consistently, expression of N-cadherin and Vimentin were increased. Recovery to normoxia reversed the changes in the mRNA levels of these EMT markers (Figure 2C). Immunoblotting analysis was used to detect the expression of E-cadherin, N-cadherin and Vimentin on protein level, showing a consistent expression profile of these markers (Figure 2D). These data indicate that induction of EMT by hypoxia is reversible.

Cancer cells underlying EMT have been documented to possess a high motility. Thus, we evaluated the effect of hypoxia on HCC motility. Our data showed that ability of migration and invasion was significantly increased when HCC cells were cultured in hypoxic condition, as compared with HCC cells cultured in normoxic condition (Figure 2E). Moreover, ability of migration and invasion was significantly decreased when hypoxia-cultured HCC cells were returned to normoxic condition. In addition, we also investigated the influence of hypoxia on colony formation capacity and cell cycle progression of HCC cells, finding G_0_/G_1_ cell cycle arrest (Additional file 4: Figure S3) and decreased numbers of colony under this condition (Additional file 5: Figure S4). These data indicate that hypoxia can induce EMT and increase capacity of migration and invasion in HCC cells

### CoCl_2_-induced HIF-1α stabilization promotes SNAI1 expression, EMT and invasion capacity of HCC cells

**Figure 3:**
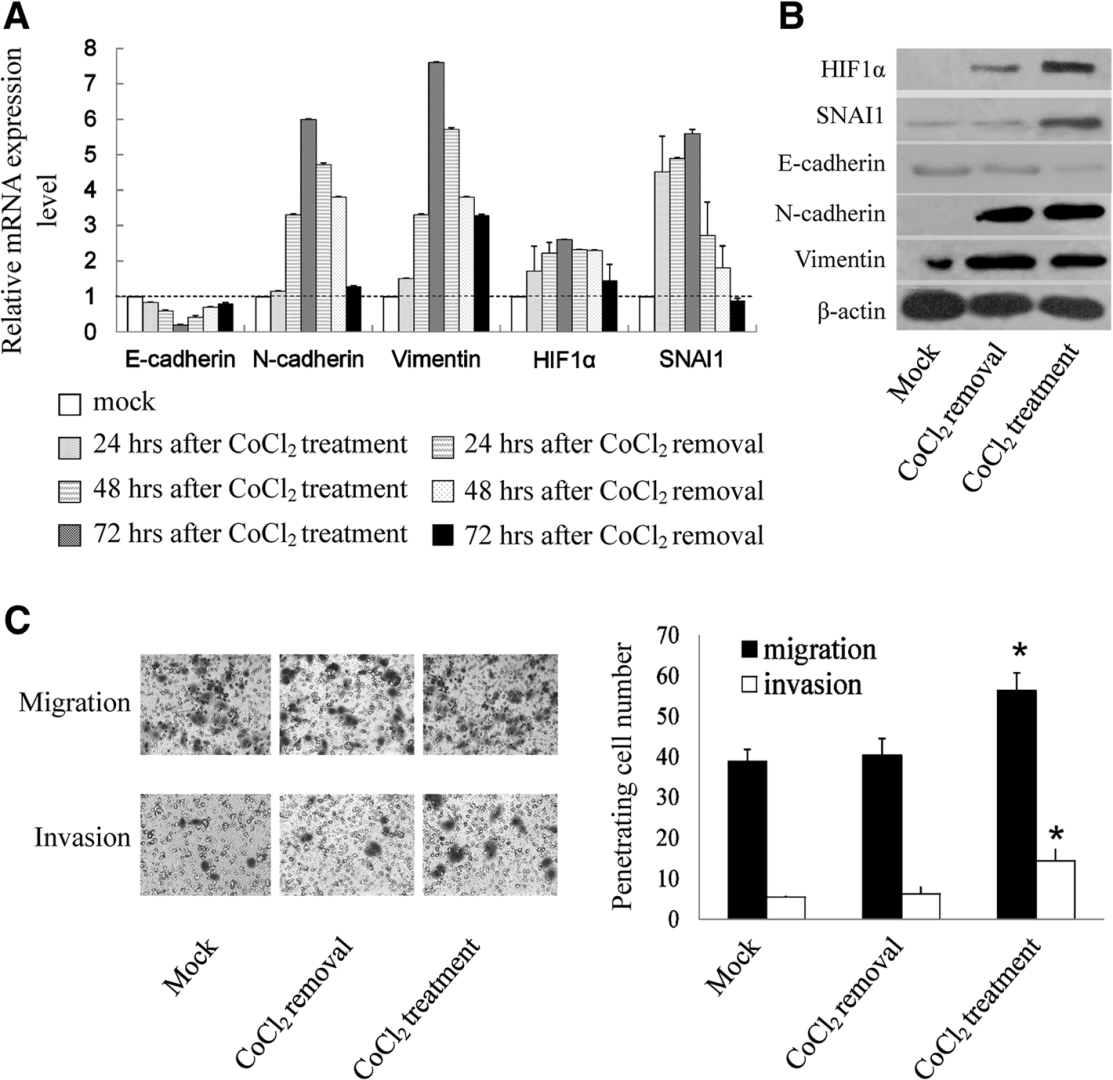
**CoCl**_**2**_**-induced HIF-1α stabilization promotes SNAI1 expression, EMT and invasion capacity of HCC cells.** (**A**) mRNA expression level of E-cadherin, N-cadherin, Vimentin, HIF-1α and SNAI1 was determined in SMMC-7721 cells with and without CoCl_2_ exposure (100 μM) by qPCR. GAPDH was used as endogenous control and mRNA level of untreated SMMC-7721cells was used as control. Data were shown as mean ± SD of three independent experiments. (**B**) Protein expression of E-cadherin, N-cadherin, Vimentin, HIF-1α and SNAI1 in SMMC-7721 cells with or without CoCl_2_ treatment (100 μM) was determined by immunoblotting. β-actin was used as endogenous reference. (**C**) The numbers of invasive and migrating SMMC-7721 cells with and without CoCl_2_ exposure (100 μM) was calculated with crystal violet staining. The average numbers of ten random microscopic fields (×400) was recorded in each experiment. Data was shown as mean ± SD of three independent experiments. Representative images of each group were shown.

We intended to reveal the molecular mechanisms by which hypoxia induced HCC cells to undergo EMT. Thus, we treated SMMC-7721 cells with CoCl_2_ (100 μM) to prevent the degradation of HIF-1α and estimated the mRNA expression of E-cadherin, N-cadherin and Vimentin in CoCl_2_-treated cells. The data demonstrated the same changes in mRNA and protein levels of these genes as those found in hypoxia-treated SMMC-7721 cells (Figure 3A and B). After removal of CoCl_2_, expression of these genes was returned to the basic level. Interestingly, mRNA and protein levels of SNAI1 was elevated in SMMC-7721 cells after treatment with CoCl_2_ and was returned to the basic level after removal of CoCl_2_ (Figure 3A and B).

CoCl_2_-induced HIF-1α stabilization also affected the biological behaviors of SMMC-7721 cells. Cells treated with CoCl_2_ were shown to have an increased ability of migration and invasion, as compared with controls. After removal of CoCl_2_, the increased ability of migration and invasion was returned to normal (Figure 3C). These data indicate that HIF-1α stabilization is able to promote SNAI1-involved EMT in HCC cells and facilitate their invasion.

### HIF-1α silencing in HCC cells inhibits SNAI1-mediated EMT and invasion under CoCl_2_ treatment

**Figure 4:**
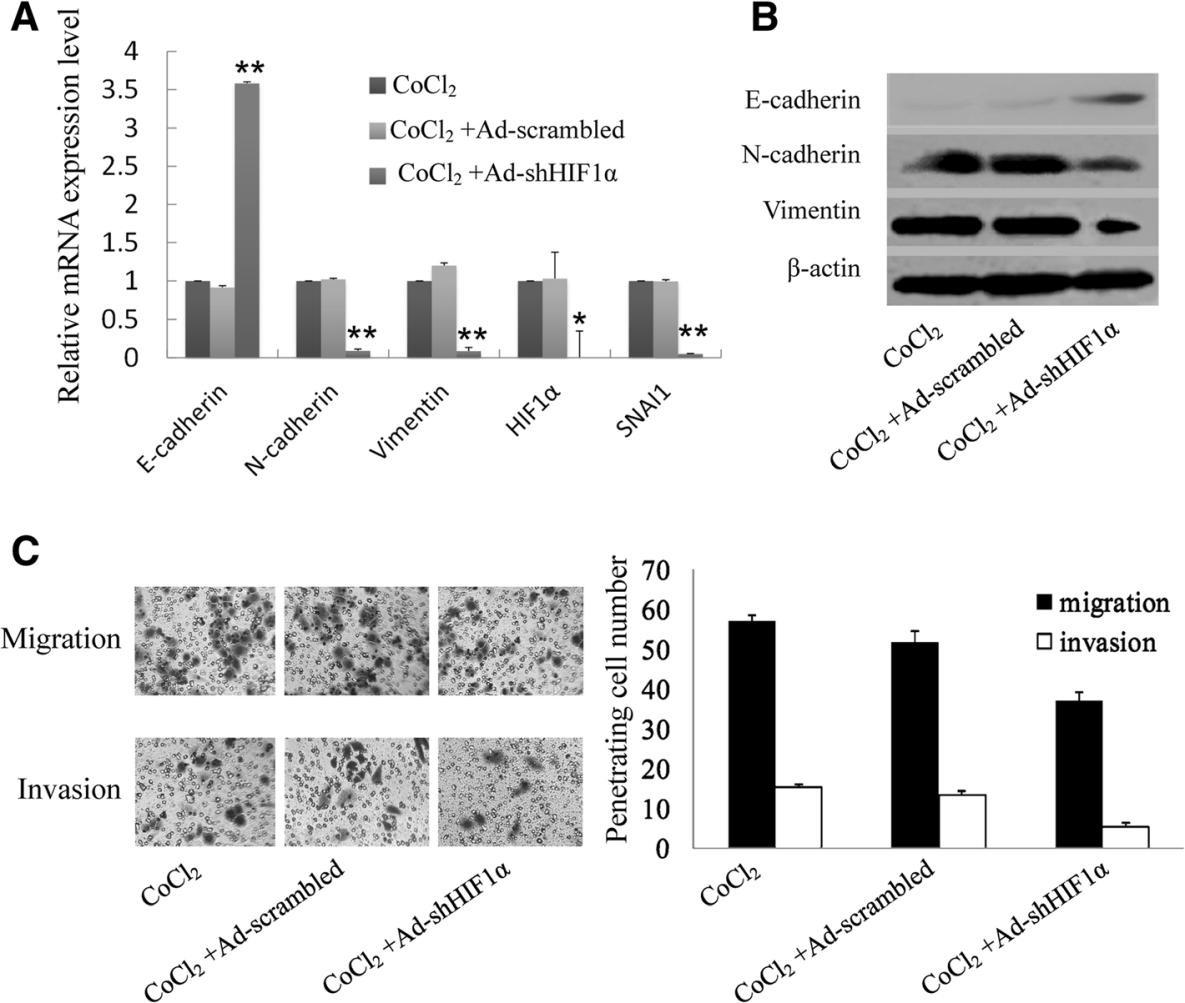
**HIF-1α silencing in HCC cells inhibits SNAI1-mediated EMT and invasion under CoCl**_**2 **_**treatment.** (**A**) mRNA levels of HIF-1α, SNAI1, E-cadherin, N-cadherin and Vimentin were determined in CoCl_2_-treated SMMC-7721 cells infected with Ad-scrambled or Ad-shHIF1α (10 MOI) by qRT-PCR. GAPDH was used as endogenous reference and mRNA level of untreated SMMC-7721cells was used as standard. Data were shown as mean ± SD of three independent experiments. (**B**) Protein levels of E-cadherin, N-cadherin and Vimentin in CoCl_2_-treated SMMC-7721 cells infected with Ad-scrambled or Ad-shHIF1a was determined by immunoblotting. β-actin was used as endogenous reference. (**C**) The numbers of invasive and migrating CoCl_2_-treated SMMC-7721 cells infected with Ad-scrambled or Ad-shHIF1α was calculated with crystal violet staining. The average numbers of ten random microscopic fields (×400) was recorded in each experiment. Data was shown as mean ± SD of three independent experiments. Representative images of each group were shown.

To confirm the role of HIF-1α in SNAI1 expression and EMT induction, we knockdowned expression of HIF-1α in HCC cells by adenoviral vector expression shRNA against HIF-1α and study expression of EMT related genes. Our result showed that expression of SNAI1, N-cadherin and Vimentin was reduced in CoCl_2_-treated HCC cells after infection with Ad-shHIF1α, whereas E-cadherin was increased in CoCl_2_-treated HCC cells after infection with Ad-shHIF1α (Figure 4A and B). Silencing of HIF-1α could significantly reduce migration and invasion of HCC cells (Figure 4C). This data indicated that HIF-1α played a key role in hypoxia-induced EMT and cell migration as well as invasion.

### HIF-1α promotes transcription of SNAI1 under hypoxic condition

**Figure 5:**
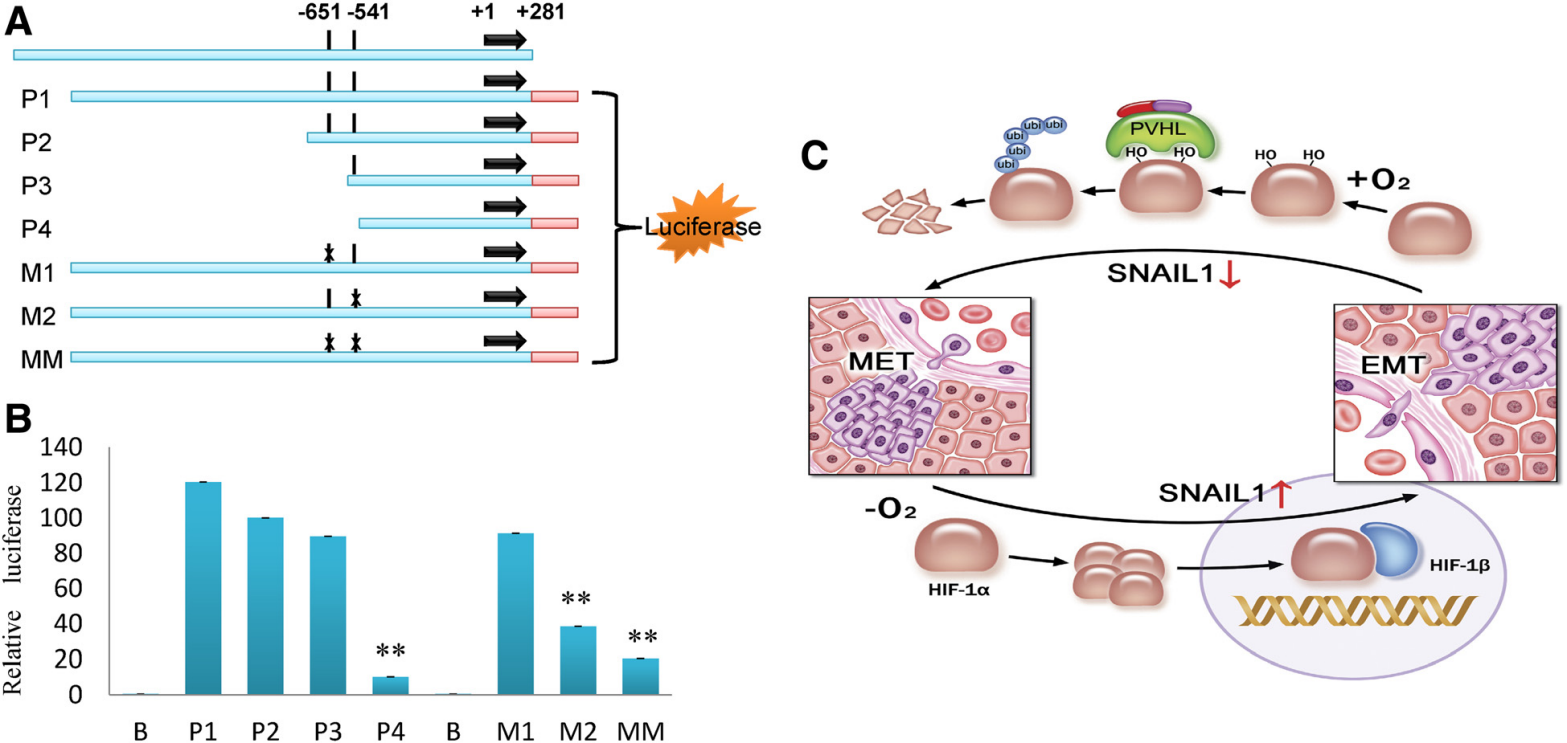
**HIF-1α promotes transcription of SNAI1 under hypoxia condition.** (**A**) Schematic diagram of SNAI1 promoter-luciferase construct was shown with the location of HRE. (HRE: hypoxia response element) (**B**) SMMC-7721 cells were transfected with pGL3-basic vector or a series of pGL3 vectors containing truncated SNAI1 promoters or promoters with mutated HRE (P1, P2, P3, P4, M1, M2 and MM) along with renilla luciferase expression vector. Luciferase assay was performed after 2 days. The firefly luciferase activity was normalized by renilla luciferase activity. Data were shown as mean ± SD of three independent experiments. (**C**) A model was shown for effect of HIF-1α on SNAI1-mediated EMT in HCC.

To further elucidate the mechanism underlying HIF-1α triggered SNAI1 upregulation, we screened the sequence of SNAI1 promoter by bioinformatics analysis and found two putative HIF-1α responsive elements (HREs) localized in −651 and −541 of this region (Figure 5A). The existence of HERs in SNAI1 promoter raised the possibility that HIF-1α may regulated the transcription of SNAI1 by binding these sites. Therefore, we constructed a series of reporter vectors (P1-P4) where luciferase expressions were driven by SNAI1 promoters of various lengths (Figure 5A). Luciferase activity was quantified after SNAI1 promoter-driven vectors were transfected into CoCl_2_-pretreated HCC cells. Our results showed that short form of SNAI1 promoters containing two HRE or one HRE at −541 had a slightly reduced activity, whereas the shortest form of SNAI1 promoter without HRE sites almost lost its activity (Figure 5B).

To further confirm the regulatory role of −541 HRE in SNAI1 transcription, we generated several reporter vectors containing mutant HRE sites (M1, M2 and MM) (Figure 5A). Our results showed that SNAI1 gene promoter only containing mutant HRE site at −651 had slight reduced activity, whereas SNAI1 gene promoter only containing mutant HRE site at −541 had significant reduced activity. SNAI1 gene promoter containing mutant HRE sites at −651 and −541 had the lowest activity (Figure 5B). These data indicate that HRE at −541 site plays an important role in transcription of SNAI1 by HIF-1α. Taken together, we conclude that HIF-1α promotes the expression of SNAI1 through recognizing the HRE in its upstream region.

## Discussion

In this study, we found the increased expression of HIF-1α in HCC samples obtained from surgical resection. Ectopic expression profile of HIF-1α is correlated with poor prognosis and enhanced HCC invasion and metastasis. Further analysis showed that increased HIF-1α level was associated with loss of E-cadherin and overexpression of SNAI1, N-cadherin and Vimentin. Our data suggest that hypoxia may induce EMT of cancer cells in HCC.

To test this hypothesis, we treated HCC cells under hypoxic condition. We found that hypoxia could induce EMT in HCC cells and enhance cell migration and invasion. Furthermore, we found that induction of EMT by hypoxia was reversible when cells were returned to normoxic condition. In addition, we confirmed that hypoxia led to G_0_/G_1_ arrest of HCC cells, which is coincident with previous reports [37-41]. CoCl_2_-induced HIF-1α stabilization also promoted EMT in HCC cells. And shRNA-mediated HIF-1α suppression was able to prevent EMT. All these data confirm that HIF-1α is an important stimulatory factor of EMT process in HCC cells.

The downstream target genes regulated by HIF-1α are involved in angiogenesis, hypoxic metabolism, cancer cell survival and invasion [10,42-46]. HIF-1α is also documented to be an upstream regulatory factor of many EMT modulators, such as SNAI1, twist, Zeb1, SIP1 and LOX [47]. Recent studies revealed that HIF-1α-induced LOX overexpression promoted the metastasis of breast cancers in a mouse model and was correlated with poor prognosis of ER negative patients [48]. Response to hypoxia was also utilized in tumor therapy in the field of gene therapy. Oncolytic adenoviruses were shown to selectively and effectively proliferate in cancer cells, when its E1B gene expression was driven by HRE-modulated promoters [49]. It is well demonstrated that SNAI1 is an inducer of EMT and it plays an important role in induction of EMT in HCC cells [50,51]. Thus, we investigated the potential effect of HIF-1α on SNAI1 expression.

Bioinformatics analysis on SNAI1 promoter identified two putative HREs, providing the possibility that HIF-1α can directly bind these sites and promoter SNAI1 transcription. Using luciferase report systems, we determined that vectors containing either of these two HREs had high luciferase activity in CoCl_2_-treated HCC cells. The vector containing -651 bp HRE apparently had higher luciferase expression than that harboring -541 bp HRE. Previous study has shown that hypoxia could induce Snail expression during EMT [52]. Recently, Luo et al. demonstrated that HIF could directly regulated mouse Snail expression [53]. Furthermore, it was reported that hypoxia induced EMT in melanoma via regulation of Snail by HIF-2α [54]. So we confirmed that HIF-1α promoted the transcription of one of central EMT-inducer, SNAI1, in hypoxia-simulating HCC model.

Collectively, we present our hypothesis of hypoxia participating in EMT of HCC cells (Figure 5C). In hypoxic conditions of the primary solid tumor, the oxygen required for proline hydroxylase activity is absent. HIF-1α in turn escapes proteolysis, allowing for its entry into the nucleus. Then, it can dimerize with HIF-1β to form the active transcription-stimulating complex, which binds HRE in SNAI1 promoter to promote SNAI1 expression. The tumor cells acquire mesenchymal phenotype, disseminate from the primary tumors, penetrate extracellular matrix (ECM) and enter blood or lymphatic vessels. As soon as some of these tumors cells penetrate ECM and enter the parenchyma of targeting tissues or organs on the condition of reoxygenation, HIF-1α is rapidly oxidized at either or both of two proline residues by a proline hydroxylase enzyme. This hydroxylation permits the binding of the von hippel-landau protein (pVHL) to HIF-1α. Once bound, HIF-1α is polyubiquitinated and subsequently degraded in the proteasome. Subsequently, the mesenchymal tumor cells undergo MET. HIF-1α may play a central role in EMT induced by hypoxia. HIF-1α-SNAI1-EMT may be one of the key signal pathways.

## Conclusion

We found that in HCC, hypoxia-induced HIF-1α stabilization promoted SNAI1-mediated EMT process, and led to the enhanced HCC invasion and metastasis and poor prognosis of patients. Further investigations to illuminate the intimate mechanisms of hypoxia and reoxygenation inducing solid tumors metastasis may lead to new molecular therapies besides conventional treatments against malignant solid tumors.

## Competing interests

The authors declare that they have no competing interests.

## Authors’ contributions

CQ, JD and XL designed the research studies. LZ, GH, YZ, YJ, JS, JZ, QW and XF carried out the experiments; CQ, LZ, JL and QW analyzed and interpreted the data; ZL, JL and CQ wrote the draft of the manuscript. All authors read and approved of the final manuscript.

## Pre-publication history

The pre-publication history for this paper can be accessed here:

http://www.biomedcentral.com/1471-2407/13/108/prepub

## Supplementary Material

Additional file 1: Table S1 Expression level of HIF-1α, HIF-2α, SNAI1 and Twist in HCC samples. **Table S2.** Correlation between HIF-1α, SNAI1, E-cadherin, N-cadherin and Vimentin in HCC samples. **Table S3.** Expression level of E-cadherin, N-cadherin and Vimentin in HCC samples. **Table S4.** Clinical significance of HIF-1α and SNAI1 expression in HCC sample. **Table S5.** Clinical significance of EMT markers in HCC sample. **Table S6.** Sequences of primers used in qPCR. Click here for file

Additional file 2: Figure S1 Morphological changes of hypoxia and reoxygenation-treated HepG2 cells were recorded by light microscope (×200). Click here for file

Additional file 3: Figure S2 Expression of N-cadherin and Vimentin in SMMC-7721 cells by Immunofluorescent staining. Immunofluorescent analysis of N-cadherin and Vimentin was performed in hypoxically cultured SMMC-7721 (×200). Click here for file

Additional file 4: Figure S3 Cell cycle was analyzed in HCC cells in hypoxia and reoxygenation conditions. Click here for file

Additional file 5: Figure S4 Clone formation efficiency was analyzed in HCC cells in hypoxia and reoxygenation conditions. Click here for file
