# Supplementary material for: Hypoxia induces epithelial-mesenchymal transition via activation of SNAI1 by hypoxia-inducible factor -1α in hepatocellular carcinoma
Source: BMC Cancer. 2013 Mar 9;13:108. doi: 10.1186/1471-2407-13-108 (PMC3614870; doi:10.1186/1471-2407-13-108)
Supplement: Additional file 1: Table S1 — Expression level of HIF-1α, HIF-2α, SNAI1 and Twist in HCC samples. Table S2. Correlation between HIF-1α, SNAI1, E-cadherin, N-cadherin and Vimentin in HCC samples. Table S3. Expression level of E-cadherin, N-cadherin and Vimentin in HCC samples. Table S4. Clinical significance of HIF-1α and SNAI1 expression in HCC sample. Table S5. Clinical significance of EMT markers in HCC sample. Table S6. Sequences of primers used in qPCR. [file 1471-2407-13-108-S1.doc]

**Table S1. Expression level of HIF-1α, HIF-2α, SNAI1 and Twist in HCC samples**

|  |  | SNAI1 | | P value | Twist | | P value |
| --- | --- | --- | --- | --- | --- | --- | --- |
|  |  | + | - |  | + | - |  |
| HIF-1α | + | 33 | 10 |  | 22 | 21 |  |
|  | - | 6 | 17 | <0.01 | 6 | 17 | <0.05 |
| HIF-2α | + | 18 | 4 |  | 18 | 4 |  |
|  | - | 10 | 34 | <0.01 | 34 | 10 | <0.01 |

**Table S2. Correlation between HIF-1α, SNAI1, E-cadherin, N-cadherin and Vimentin in HCC samples**

|  |  | E-cadherin | | P value | N-cadherin | | P value | Vimentin | | P value |
| --- | --- | --- | --- | --- | --- | --- | --- | --- | --- | --- |
|  |  | + | - |  | + | - |  | + | - |  |
| HIF-1α | + | 10 | 33 |  | 34 | 9 |  | 18 | 25 |  |
|  | - | 18 | 5 | <0.01 | 7 | 16 | <0.01 | 0 | 23 | <0.01 |
| SNAI1 | + | 8 | 31 |  | 33 | 6 |  | 14 | 25 |  |
|  | - | 20 | 7 | <0.01 | 8 | 19 | <0.01 | 4 | 23 | 0.059 |

**Table S3. Expression level of E-cadherin, N-cadherin and Vimentin in HCC samples**

|  |  | N-cadherin | | P value | Vimentin | | P value |
| --- | --- | --- | --- | --- | --- | --- | --- |
|  |  | + | - |  | + | - |  |
| E-cadherin | + | 10 | 18 |  | 1 | 27 |  |
|  | - | 31 | 7 | <0.01 | 17 | 21 | <0.01 |

**Table S4. Clinical significance of HIF-1α and SNAI1 expression in HCC sample**

|  |  | HIF-1α | | P value | SNAI1 | | P value |
| --- | --- | --- | --- | --- | --- | --- | --- |
|  |  | + | - |  | + | - |  |
| Gender | Male | 39 | 21 | 1.00 | 34 | 26 | 0.388 |
|  | Female | 4 | 2 |  | 5 | 1 |  |
| Age | <=55 | 29 | 15 | 1.00 | 27 | 17 | 0.595 |
|  | >55 | 14 | 8 |  | 12 | 10 |  |
| HBsAg | - | 5 | 2 | 1.00 | 4 | 3 | 1.000 |
|  | + | 38 | 5 |  | 35 | 24 |  |
| HBV-DNA | ＜10^3^ | 25 | 12 | 0.576 | 18 | 19 | 0.150 |
|  | 10^3^-10^5^ | 8 | 3 |  | 8 | 3 |  |
|  | ＞10^5^ | 10 | 8 |  | 13 | 5 |  |
| Child-Pugh classification | A | 42 | 22 | 1.00 | 39 | 25 | 0.217 |
|  | B |  |  |  |  |  |  |
|  | C | 1 | 0 |  | 0 | 1 |  |
| Severity of cirrhosis | no | 19 | 10 | 0.016* | 15 | 14 | 0.148 |
|  | mild | 14 | 3 |  | 14 | 3 |  |
|  | moderate | 5 | 6 |  | 6 | 5 |  |
|  | severe | 5 | 4 |  | 4 | 5 |  |
| AFP | ＜20 ng/ml | 14 | 7 | 0.860 | 12 | 9 | 0.826 |
|  | ≥20 ng/ml | 29 | 16 |  | 27 | 11 |  |
| Tumor thrombus | - | 41 | 22 | 0.89 | 38 | 25 | 0.67 |
|  | + | 2 | 1 |  | 1 | 2 |  |
| Tumor capsule | - | 37 | 19 | 0.730 | 34 | 22 | 0.729 |
|  | + | 6 | 4 |  | 5 | 5 |  |
| Edmondson pathological classification | Ⅰ | 6 | 10 | 0.001* | 11 | 5 | 0.012* |
|  | Ⅱ | 10 | 11 |  | 11 | 10 |  |
|  | Ⅲ | 23 | 1 |  | 14 | 10 |  |
|  | Ⅳ | 4 | 0 |  | 3 | 1 |  |
| TNM staging | Ⅰ | 30 | 20 | 0.009* | 25 | 25 | 0.012* |
|  | Ⅱ | 5 | 1 |  | 6 | 0 |  |
|  | Ⅲ | 8 | 2 |  | 8 | 2 |  |
| Number of tumor nodule | 1 | 32 | 18 | 0.769 | 29 | 21 | 0.444 |
|  | 2 | 7 | 4 |  | 8 | 3 |  |
|  | 3 | 4 | 1 |  | 2 | 3 |  |
| Maximum tumor size (cm) | ≤5 | 17 | 17 | 0.008* | 9 | 25 | 0.005* |
|  | >5 | 26 | 6 |  | 30 | 2 |  |

**Table S5. Clinical significance of EMT markers in HCC sample**

|  |  | E-cadherin | | p | N-cadherin | | p | Vimentin | | p |
| --- | --- | --- | --- | --- | --- | --- | --- | --- | --- | --- |
|  |  | + | - |  | + | - |  | + | - |  |
| Gender | Male | 26 | 34 | 1.00 | 38 | 22 | 0.666 | 17 | 43 | 1.000 |
|  | Female | 2 | 4 |  | 3 | 3 |  | 1 | 5 |  |
| Age | <=55 | 17 | 27 | 0.379 | 25 | 19 | 0.209 | 8 | 36 | 0.879 |
|  | >55 | 11 | 11 |  | 16 | 6 |  | 10 | 12 |  |
| HBsAg | - | 1 | 6 | 0.224 | 5 | 2 | 0.701 | 2 | 5 | 1.000 |
|  | + | 27 | 32 |  | 36 | 23 |  | 16 | 43 |  |
| HBV-DNA | ＜10^3^ | 15 | 22 | 0.939 | 23 | 14 | 0.991 | 10 | 27 | 0.998 |
|  | 10^3^-10^5^ | 5 | 6 |  | 7 | 4 |  | 3 | 8 |  |
|  | ＞10^5^ | 8 | 10 |  | 11 | 7 |  | 5 | 13 |  |
| Child-Pugh classification | A | 26 | 38 | 1.000 | 40 | 24 | 1.000 | 18 | 46 | 1.000 |
|  | B |  |  |  |  |  |  |  |  |  |
|  | C | 1 | 0 |  | 1 | 0 |  | 0 | 1 |  |
| Severity of cirrhosis | no | 15 | 14 | 0.275 | 16 | 13 | 0.614 | 7 | 22 | 0.724 |
|  | mild | 5 | 12 |  | 12 | 5 |  | 6 | 11 |  |
|  | moderate | 3 | 8 |  | 8 | 3 |  | 2 | 9 |  |
|  | severe | 5 | 4 |  | 5 | 4 |  | 3 | 6 |  |
| AFP | ＜20 ng/ml | 10 | 11 | 0.560 | 15 | 6 | 0.287 | 4 | 17 | 0.305 |
|  | ≥20 ng/ml | 18 | 27 |  | 26 | 19 |  | 14 | 31 |  |
| Tumor thrombus | - | 27 | 36 | 1.00 | 40 | 23 | 0.552 | 16 | 47 | 0.178 |
|  | + | 1 | 2 |  | 1 | 2 |  | 2 | 1 |  |
| Tumor capsule | - | 21 | 35 | 0.083 | 36 | 20 | 0.485 | 18 | 38 | 0.048* |
|  | + | 7 | 3 |  | 5 | 5 |  | 0 | 10 |  |
| Edmondson pathological classification | Ⅰ | 16 | 1 | 0.001* | 13 | 3 | 0.003* | 6 | 27 | 0.001* |
|  | Ⅱ | 8 | 13 |  | 17 | 4 |  | 8 | 13 |  |
|  | Ⅲ | 3 | 21 |  | 18 | 6 |  | 1 | 6 |  |
|  | Ⅳ | 1 | 3 | 3 | 1 | 3 | 1 |  |  |  |
| TNM staging | Ⅰ | 25 | 25 | 0.012* | 28 | 22 | 0.009* | 5 | 45 | 0.001* |
|  | Ⅱ | 1 | 5 |  | 5 | 1 |  | 4 | 2 |  |
|  | Ⅲ | 2 | 8 |  | 8 | 2 |  | 9 | 1 |  |
| Number of tumor nodule | 1 | 25 | 25 | 0.036* | 27 | 23 | 0.041* | 4 | 46 | 0.012* |
|  | 2 | 3 | 8 |  | 10 | 1 |  | 10 | 1 |  |
|  | 3 | 0 | 5 |  | 4 | 1 |  | 4 | 1 |  |
| Maximum tumor size (cm) | ≤5 | 20 | 14 | 0.005* | 18 | 16 | 0.013* | 6 | 38 | 0.030* |
|  | >5 | 8 | 24 |  | 23 | 9 |  | 12 | 10 |  |

**Table S6. Sequences of primers used in qPCR**

| gene | sequence | | Product length |
| --- | --- | --- | --- |
| E-Cadherin | Forward primer | 5’-AGAGAACGCATTGCCACATACA-3’ | 242bp |
|  | Reverse primer | 5’-TAAGCGATGGCGGCATTGTA-3’ |  |
| N-Cadherin | Forward primer | 5’-AGGCTTCTGGTGAAATCGCA-3’ | 213bp |
|  | Reverse primer | 5’-GTGGAAAGCTTCTCACGGCA-3’ |  |
| Vimentin | Forward primer | 5’-AGGCTTCTGGTGAAATCGCA-3’ | 213bp |
|  | Reverse primer | 5’-GTGGAAAGCTTCTCACGGCA-3’ |  |
| GAPDH | Forward primer | 5’-GGGGAAGGTGAAGGTCGGAG-3’ | 240bp |
|  | Reverse primer | 5’-TCTCGCTCCTGGAAGATGGTGAT-3’ |  |
| β-actin | Forward primer | 5’-CGGGAAATCGTGCGTGACAT-3’ | 257bp |
|  | Reverse primer | 5’-TTGCGGATGTCCACGTCACAC-3’ |  |
| HIF-1α | Forward primer | 5'CAAGTCACCACAGGACAGTACAGG-3' | 304bp |
|  | Reverse primer | 5' GCAAAGTTAAAGCATCAGGTTCC-3 |  |
