# Supplementary figures and images for: Hypoxia induces epithelial-mesenchymal transition via activation of SNAI1 by hypoxia-inducible factor -1α in hepatocellular carcinoma
Source: BMC Cancer. 2013 Mar 9;13:108. doi: 10.1186/1471-2407-13-108 (PMC3614870; doi:10.1186/1471-2407-13-108)

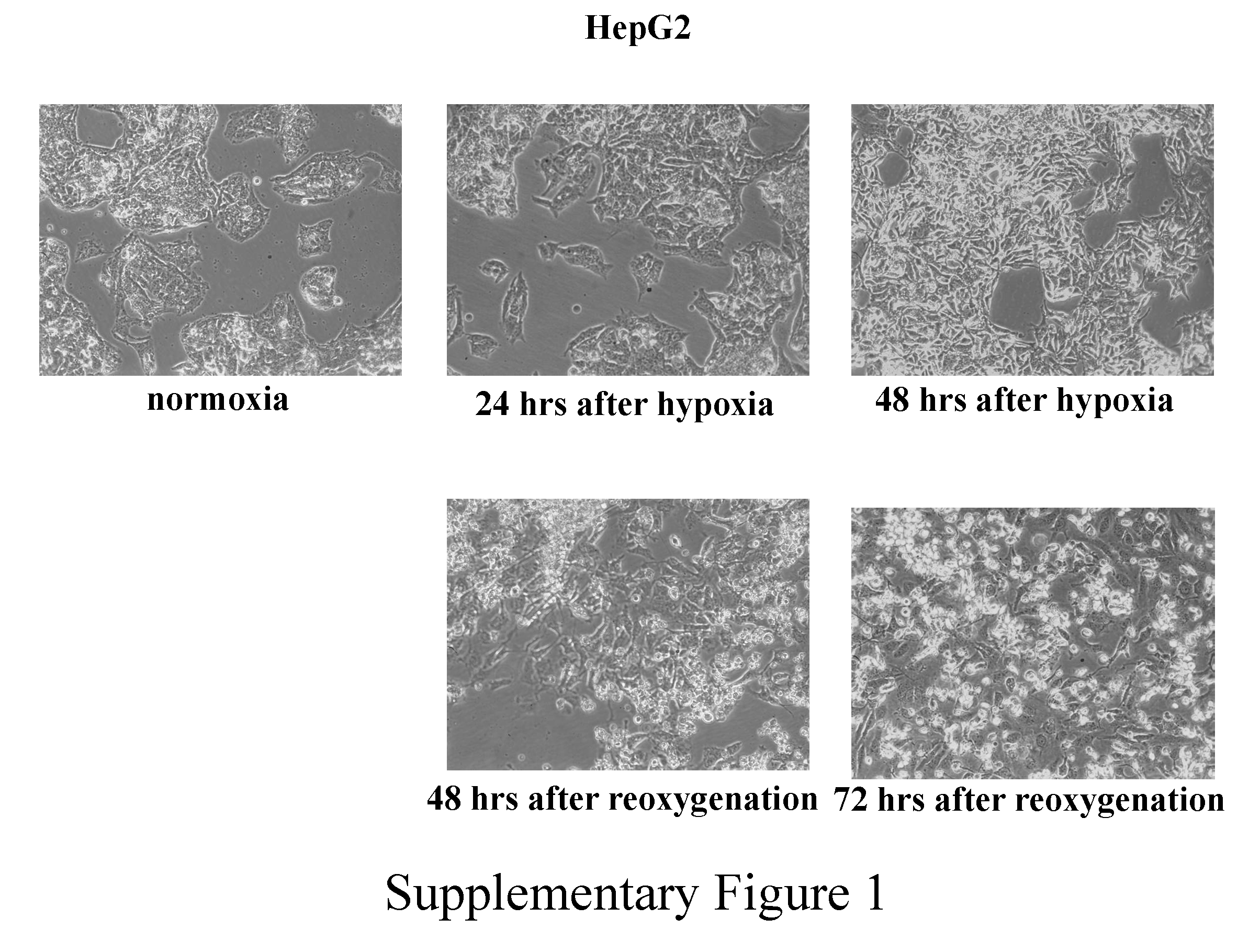

Supplement: Additional file 2: Figure S1 — Morphological changes of hypoxia and reoxygenation-treated HepG2 cells were recorded by light microscope (×200). [file 1471-2407-13-108-S2.tiff]

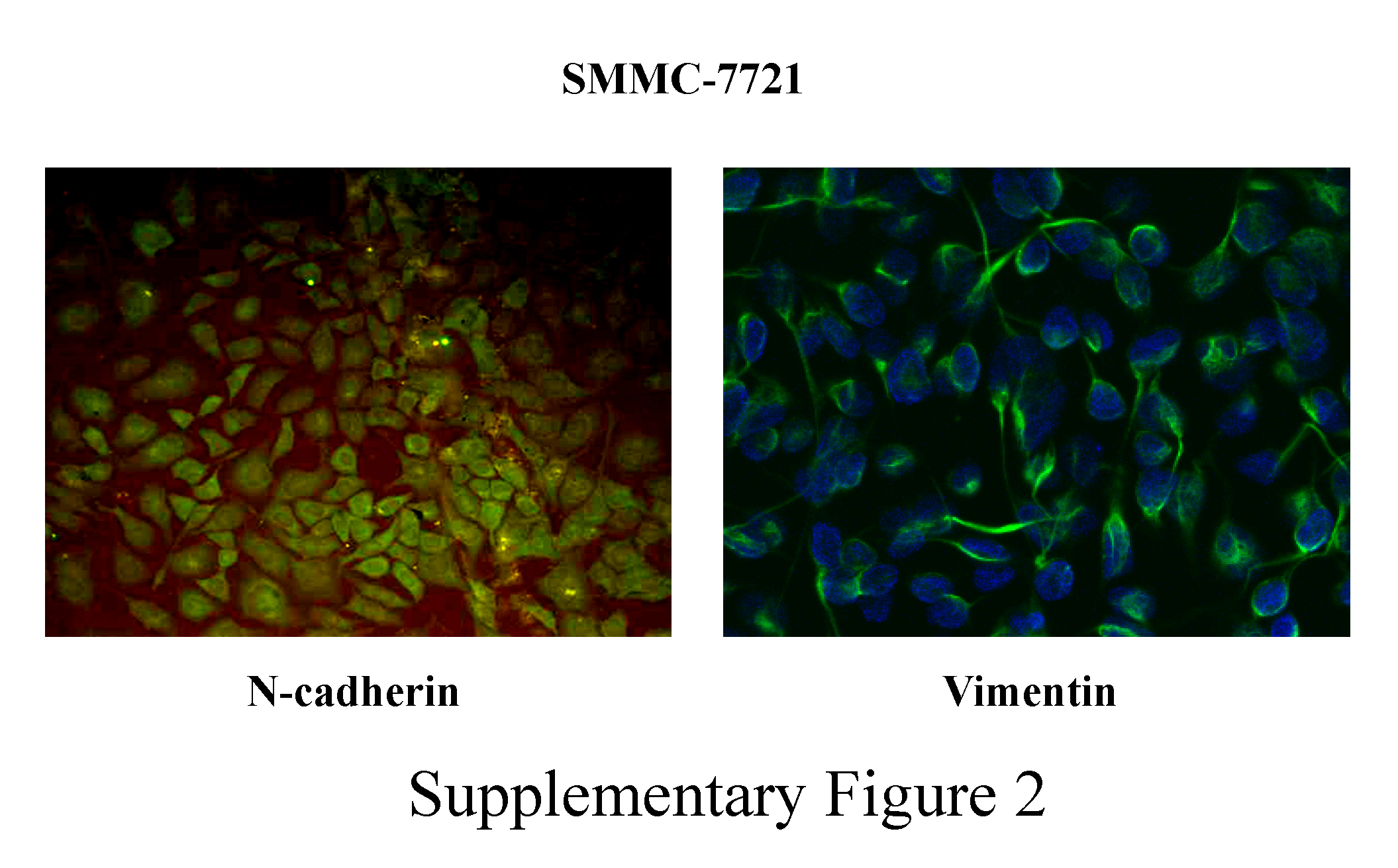

Supplement: Additional file 3: Figure S2 — Expression of N-cadherin and Vimentin in SMMC-7721 cells by Immunofluorescent staining. Immunofluorescent analysis of N-cadherin and Vimentin was performed in hypoxically cultured SMMC-7721 (×200). [file 1471-2407-13-108-S3.tiff]

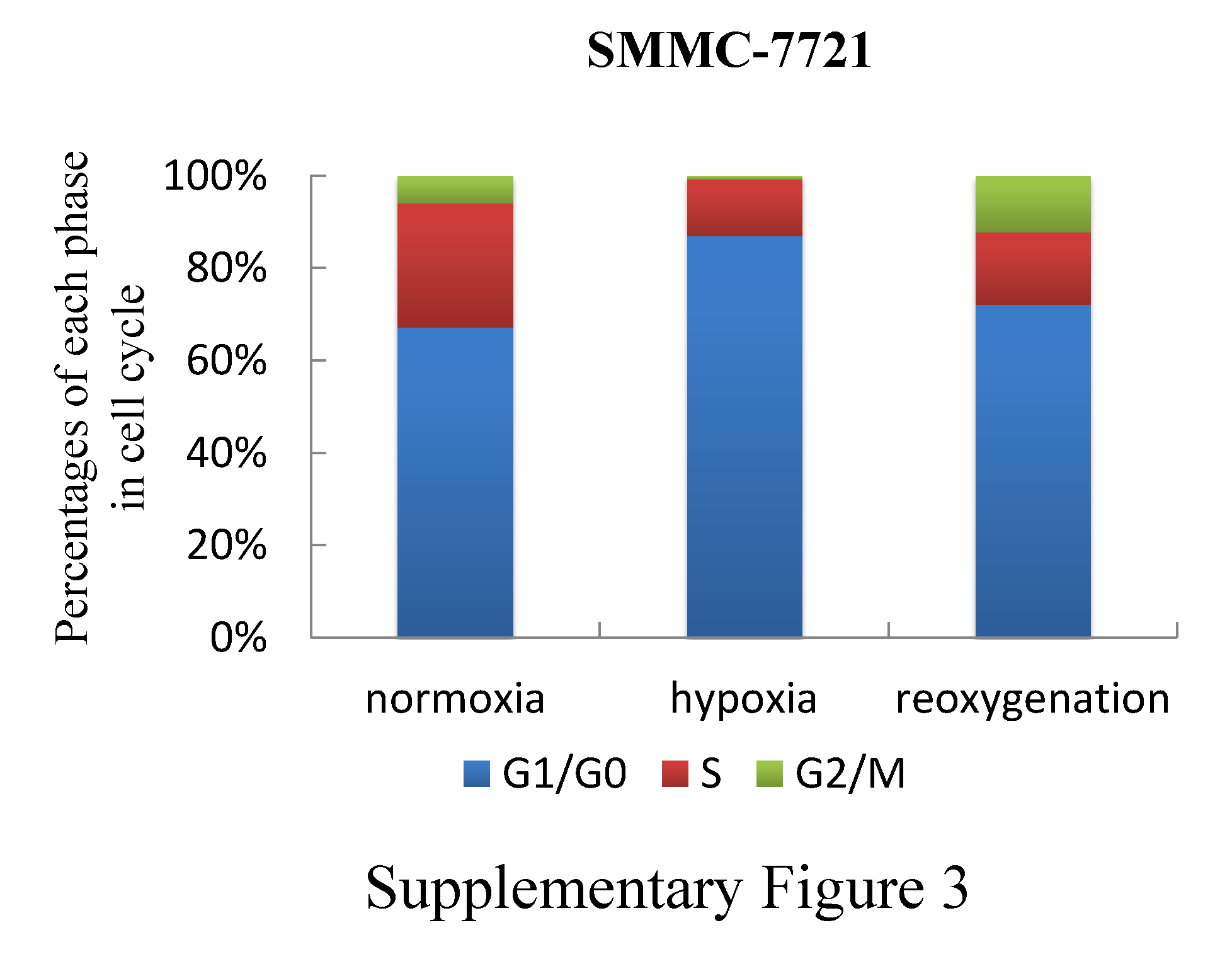

Supplement: Additional file 4: Figure S3 — Cell cycle was analyzed in HCC cells in hypoxia and reoxygenation conditions. [file 1471-2407-13-108-S4.tiff]

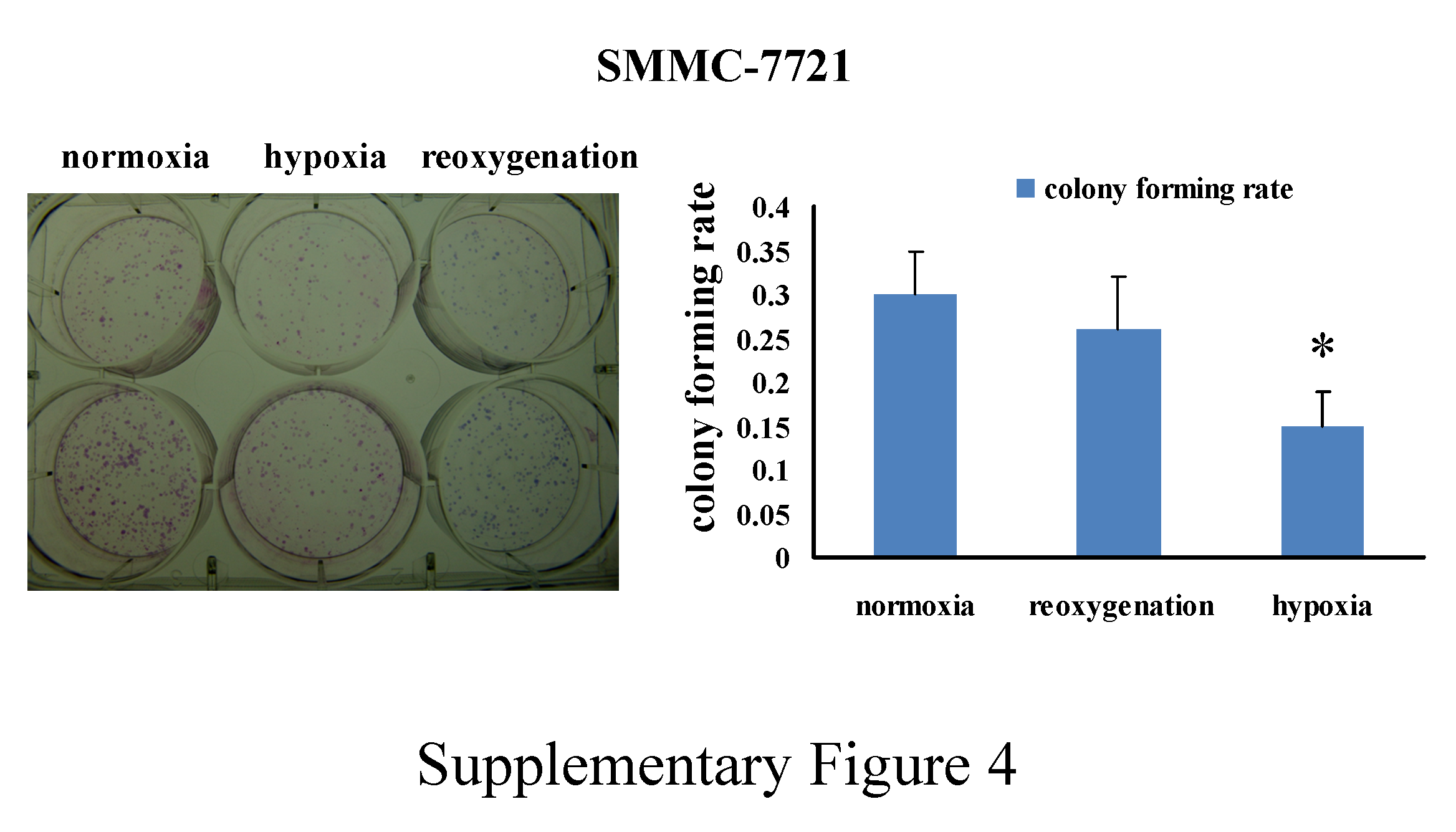

Supplement: Additional file 5: Figure S4 — Clone formation efficiency was analyzed in HCC cells in hypoxia and reoxygenation conditions. [file 1471-2407-13-108-S5.tiff]
